# Heterozygous deletion of *Cdc42bpb* does not alter ethanol behaviors in mice.

**DOI:** 10.17912/micropub.biology.000537

**Published:** 2022-03-17

**Authors:** Taylor N Foreman, Ayanna T Limaye, James W Bogenpohl

**Affiliations:** 1 Christopher Newport University; Department of Molecular Biology and Chemistry

## Abstract

The gene *Cdc42bpb* encodes a kinase with an important role in cell migration and neurodevelopment. Recent evidence suggests this gene also has an important function in the genomic response to alcohol in the brain. We tested mice with a heterozygous deletion of *Cdc42bpb* in a battery of three alcohol-related behavioral tests: loss of righting reflex, light-dark box, and two-bottle choice drinking. *Cdc42bpb* +/- mice showed no significant differences from wild type littermates in the primary output measure of any of the three tests. *Cdc42bpb* +/- mice did show a mild hyperactivity in the light-dark box, as well as some urogenital deformities.

**Figure 1. Ethanol behaviors of Cdc42bpb +/- mice:**
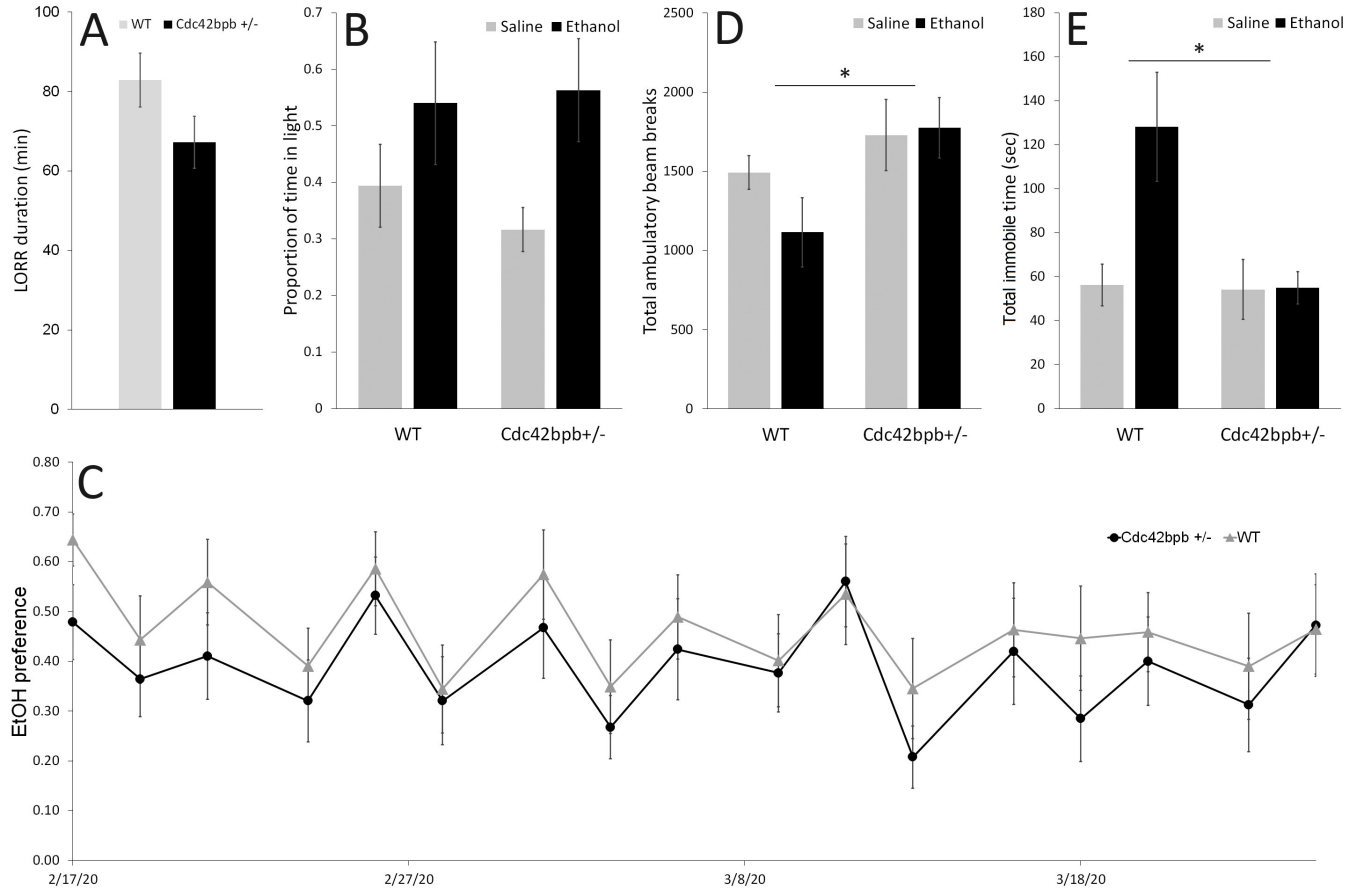
A) *Cdc42bpb* +/- mice (n=17) and wild type littermates (n=19) showed similar duration of loss of righting reflex (p=0.107). B) *Cdc42bpb* +/- mice (n=14) and wild type littermates (n=16) spent similar proportions of time in the light side of the light-dark box (p=0.924). C) *Cdc42bpb* +/- mice (n=15) and wild type littermates (n=15) showed similar ethanol preference throughout the two-bottle choice drinking study (p=0.522). D) *Cdc42bpb* +/- mice (n=14) produced significantly more ambulatory beam breaks than wild type littermates (n=16) in the light-dark box (p=0.025). E) *Cdc42bpb* +/- mice (n=14) spent significantly less time immobile than wild type littermates (n=16) in the light-dark box (p=0.025).

## Description

The gene *Cdc42bpb* encodes CDC42 binding protein kinase beta, also known as myotonic dystrophy kinase-related CDC42-binding kinase beta (MRCKβ), which is a serine/threonine kinase that phosphorylates myosin II regulatory light chain, in order to regulate cytoskeletal reorganization and cell migration (Wilkinson et al. 2005). Recent studies have found neurodevelopmental deficits in humans heterozygous for loss of function mutations in *CDC42BPB* (Chilton et al. 2020), and an epigenomics study has shown that methylation of the gene is associated with symptoms of depression (Story Jovanova et al. 2018). Importantly, a recent alcohol (ethanol) genomics study in the anterior cingulate cortex of monkeys and mice found that *CDC42BPB* ranked highly (#26 out of about 31,000 probesets) as an important mediator of ethanol’s effects on the brain, based on its ethanol-regulation of expression, correlation to ethanol phenotypes, and intra-network connectivity (Bogenpohl et al. 2019).

Given its role in neurodevelopment, involvement in mental illness, and association to ethanol, *Cdc42bpb* makes an excellent candidate gene target for studies aimed at elucidating the molecular mechanisms underpinning the development of alcohol use disorders. Therefore, we have conducted a behavioral study in mice heterozygous for a deletion of the gene (hereafter *Cdc42bpb+/-* ). Heterozygous mice were used because the gene shows haploinsufficiency in its neurodevelopmental phenotype in humans (Chilton et al. 2020), and homozygous deletion results in pre-weaning lethality in mice (Jackson Laboratory, personal communication). A battery of three behavioral tests was conducted to probe different aspects of ethanol phenotypes: a loss of righting reflex test (LORR) evaluated initial ethanol sensitivity, a light-dark box test evaluated ethanol-induced anxiolysis, and a two-bottle choice drinking study evaluated ethanol intake/preference.

In the loss of righting reflex test, *Cdc42bpb+/-* mice (n=19) and wildtype littermates (n=17) showed similar initial sensitivity to ethanol (Figure 1A). A trend was present whereby the mutant mice had a slightly shorter LORR duration, but the difference did not reach statistical significance (p=0.107, Student’s t-test). In the Light-Dark Box test, *Cdc42bpb+/-* mice (n=8 EtOH, 6 sal) and wildtype littermates (n=8 per group) showed no effect of genotype on the proportion of time spent on the light side of the box (Figure 1B; 2-way ANOVA; F(1,1)=0.009, p=0.924). In the two-bottle choice drinking study, *Cdc42bpb+/-* mice (n=15) and wildtype littermates (n=15) drank ethanol at similar rates, producing similar ethanol preferences (Figure 1C). A repeated measures ANOVA with genotype as a between-subjects factor and day as a within-subjects factor found no significant differences between ethanol preferences of the two groups (F(1,16)=0.420, p=0.522). A similar analysis of raw ethanol intake in mL instead of ethanol preference yielded similar results, showing no difference between the two groups (F(1,16)=0.688, p=0.414).

Looking at other phenotypes, the only significant behavioral difference that we were able to measure was a mild hyperactivity of *Cdc42bpb* +/- mice, as compared to wild type littermates. This was manifested as an increase in the total number of ambulatory beam breaks across genotypes (Figure 1D; 2-way ANOVA; F(1,1)=5.631, p=0.025) and a decrease in total immobile time (Figure 1E; F(1,1)= 5.623, p=0.025) in the light-dark box, particularly under the condition of ethanol treatment. A significant effect of treatment was observed for the immobile time phenotype (F(1,1)= 5.277, p=0.030), but not for ambulatory beam breaks (F(1,1)= 0.775, p=0.387). Also, a significant genotype x treatment interaction was observed for the immobile time phenotype (F(1,1)= 5.047, p=0.033) but not for ambulatory beam breaks (F(1,1)= 1.255, p=0.273). It is also worth noting that, in line with urogenital deformities reported in *CDC42BPB* +/- humans (Chilton et al. 2020), 4 out of 7 male *Cdc42bpb* +/- mice developed a partially prolapsed penis, which often became infected. Mice with this condition were removed from further participation the study and replaced when possible.

## Methods

*Mice*

All animal studies were approved by the Christopher Newport University Institutional Animal Care and Use Committee and were conducted in accordance with the NIH Guide for the Care and Use of Laboratory Animals. Male *Cdc42bpb+/-* mice on a C57BL/6NJ background were purchased from Jackson Laboratory (stock #030089) and were bred to female C57BL/6NJ mice (stock #005304) to create a breeding colony at Christopher Newport University. Mice without litters were group housed up to 4 animals per cage with *ad libitum* access to food and water. Offspring were genotyped using PCR primer sequences provided by Jackson Laboratory. At 7-10 weeks of age, *Cdc42bpb+/-* mice and wildtype littermates of both sexes began behavioral testing. Each mouse underwent each of the three behavioral tests, in the following order: loss of righting reflex, then light-dark box, then two-bottle choice, with at least one week of washout time between tests.

*Loss of righting reflex test*

*Cdc42bpb+/-* mice (n=17; 11f, 6m) and wildtype littermates (n=19; 12f, 7m) of both sexes were injected i.p. with 4 g/kg ethanol (20% v/v in 0.9% saline) and upon losing consciousness were placed on their backs in a V-shaped trough. The time elapsed between losing and regaining the ability to right themselves was quantified as an index of initial sensitivity to ethanol, with longer times representing greater sensitivity. Regaining righting reflex was defined as the ability of the mouse to flip over onto their feet twice within 60 sec. Mice that did not lose righting reflex were not included in the analysis.

*Light-dark box test*

The light-dark box test is a modified version of the open field test, where the field is split into two compartments joined by a door; one side brightly illuminated and the other darkened. The proportion of time spent on the light side of the compartment was quantified as an index of anxiety, with larger proportions representing lesser anxiety. An automated light-dark box apparatus was used that tracks the position of the mouse using a grid of infrared beams (Omnitech Electronics Inc.; Columbus, OH). *Cdc42bpb+/-* mice (n=14; 11f, 3m) and wildtype littermates (n=16; 11f, 5m) of both sexes were injected i.p. with 1.8 g/kg ethanol (20% v/v in saline), were allowed 5 minutes in their home cage for the ethanol to take effect, and were then placed into the apparatus on the light side, next to the door between compartments. Movements were then tracked for 5 minutes. Mice that did not enter the dark side of the apparatus in the 5 minutes were excluded from the analysis.

*Two-bottle choice drinking study*

*Cdc42bpb+/-* mice (n=15; 12f, 3m) and wildtype littermates (n=15; 9f, 6m) of both sexes were single housed in free cages where the standard water bottle had been replaced by two smaller graduated bottles. For the six-week duration of the study, mice were given intermittent access to ethanol on Mondays, Wednesdays, and Fridays; one bottle contained 15% ethanol in water (v/v) and the other contained water, with the left-right order of bottles switched each day. For the remainder of the week, both bottles contained water. An empty cage with bottles was included to assess the amounts of water and ethanol lost due to evaporation, and these figures were subtracted from drinking measurements each day. Drinking data were expressed as ethanol preference, which was calculated as the proportion of ethanol out of total fluids drunk each day: ethanol drunk/(ethanol+water drunk).

## Reagents

**Table:** 

**Strain**	**Genotype**	**Stock #**	**Available from**
*Cdc42bpb* +/-	C57BL/6NJ-Cdc42bpb ^em1(IMPC)J^ /J	030089	Jackson Laboratory
Wild type	C57BL/6NJ	005304	Jackson Laboratory
